# Supplementary material for: Synergistic and low adverse effect cancer immunotherapy by immunogenic chemotherapy and locally expressed PD-L1 trap
Source: Nat Commun. 2018 Jun 8;9:2237. doi: 10.1038/s41467-018-04605-x (PMC5993831; doi:10.1038/s41467-018-04605-x)
Supplement: Supplementary file 1 — Supplementary Information [file 41467_2018_4605_MOESM1_ESM.pdf]

Supporting materials for

Synergistic and Low Adverse Effect Cancer Immunotherapy by Immunogenic Chemotherapy and Locally Expressed PD-L1 Trap

Song et al.

Synergistic and Low Adverse Effect Cancer Immunotherapy by Immunogenic Chemotherapy and Locally Expressed PD-L1 Trap

Wantong Song[1,4], Limei Shen[1], Ying Wang[1], Qi Liu[1], Tyler J. Goodwin[1], Jingjing Li[2], Olekasandra Dorosheva[2], Tianzhou Liu[5], Rihe Liu[2,3,*], and Leaf Huang[1,*]

[1]Division of Pharmacoengineering and Molecular Pharmaceutics, Eshelman School of Pharmacy, University of North Carolina at Chapel Hill, Chapel Hill, NC 27599, USA.

[2]Division of Chemical Biology and Medicinal Chemistry, Eshelman School of Pharmacy, University of North Carolina at Chapel Hill, Chapel Hill, NC 27599, USA.

[3]Carolina Center for Genome Sciences, University of North Carolina at Chapel Hill, Chapel Hill, NC 27599, USA.

[4]Key Laboratory of Polymer Ecomaterials, Changchun Institute of Applied Chemistry, Chinese Academy of Sciences, Changchun, 130022, P. R. China.

[5]Department of Gastrointestinal Surgery, The Second Hospital of Jilin University, Changchun, 130041, P. R. China

* Correspondence and requests for materials should be addressed to R.L. (rliu@email.unc.edu) or to L.H. (leafh@email.unc.edu).

Supplementary file includes:

Supplementary Figures 1 to 13.

Supplementary Tables 1 to 2.

Supplementary Note 1.

Supplementary Figures

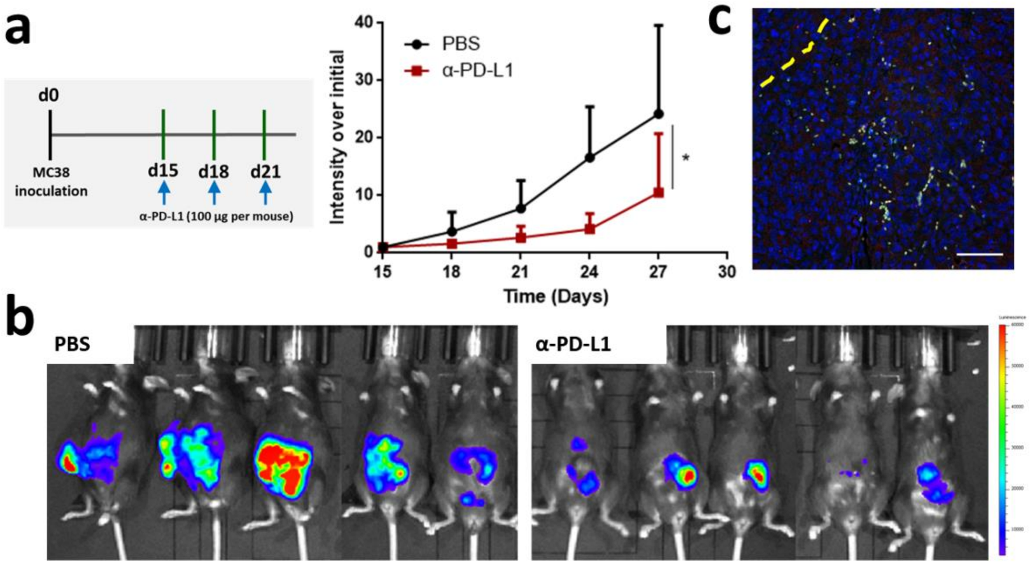

Supplementary Figure 1. Anti-PD-L1 mAb therapy in orthotopic MC38 model. **a** Treatment scheme and tumor growth curves of orthotopic MC38 tumors in PBS and α-PD-L1 treated groups (n = 5 mice per group). **b** IVIS images of the orthotopic MC38 tumors on day 27. **c** Immunofluorescence staining of orthotopic MC38 tumors in PBS group using DAPI (blue) and anti-CD3 antibody (red). Yellow dotted line indicates the border between intestinal mucosa and the orthotopic tumor. Scale bar represents 50 µm. Significant differences were assessed in **a** using two-way ANOVA. Results are presented as mean (SD). * $p < 0.05$.

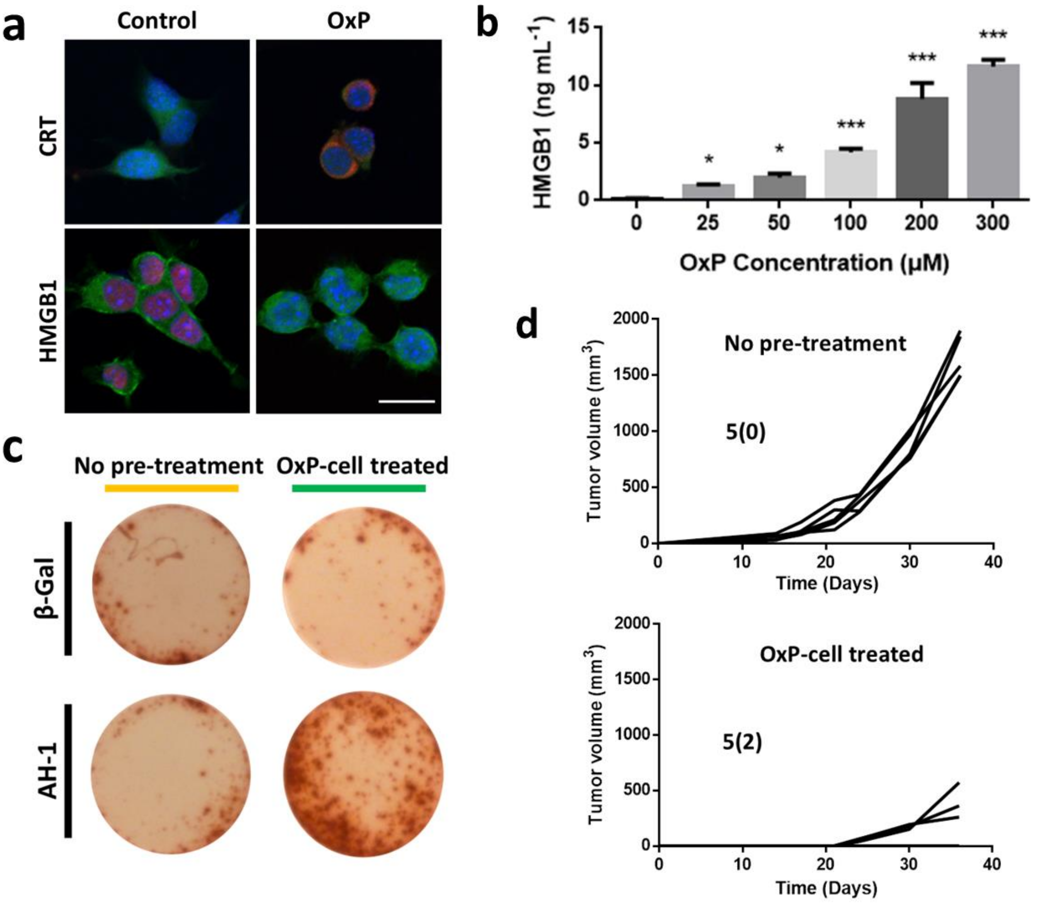

Supplementary Figure 2. OxP induced ICD in CT26-FL3 cells. (**a)** CRT exposure and HMGB1 release test of CT26-FL3 cells with or without incubation with OxP. Scale bar represents 20 μm. (**b)** ELISA detection of HMGB1 release in the supernatants of CT26-FL3 cells treated with different concentrations of OxP for 24 h (n = 3). (**c)** ELISpot test of the splenocytes of mice injected with or without OxP incubated CT26-FL3 cells. (**d)** OxP incubated CT26-FL3 cells efficiently vaccinated mice against re-challenging of CT26-FL3 cells. In the mice without pre-treatment, 0 out of 5 mice were tumor free. In the OxP incubated cells vaccinated group, 2 out of 5 mice were tumor free, and the tumor growth rate is slower. Significant differences were assessed in **b** using t test. Results are presented as mean (SD). *$p < 0.05$, ***$p < 0.001$.

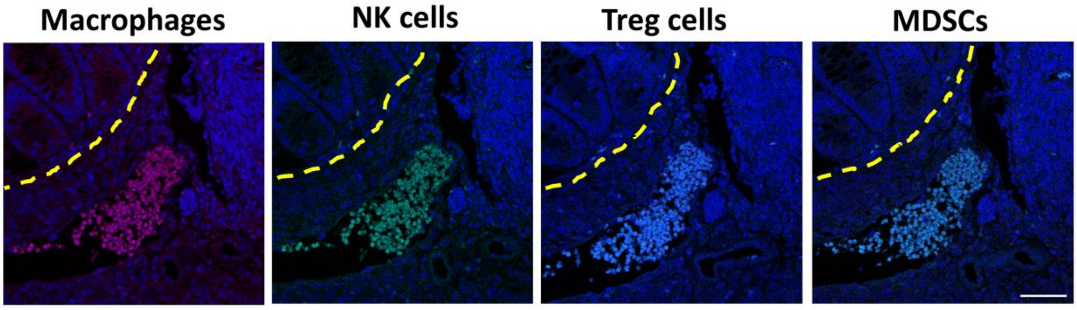

Supplementary Figure 3. Immunofluorescence staining of the orthotopic CT26-FL3 tumors after OxP treatment using DAPI (blue), anti-F4/80 (red), anti-NK1.1 (green), anti-CD4 (red) and anti-FoxP3 (green), anti-CD11b (green) and anti-Gr-1 (red). Yellow dotted line indicates the border between intestinal mucosa and the orthotopic tumor. Scale bar represents 50 μm.

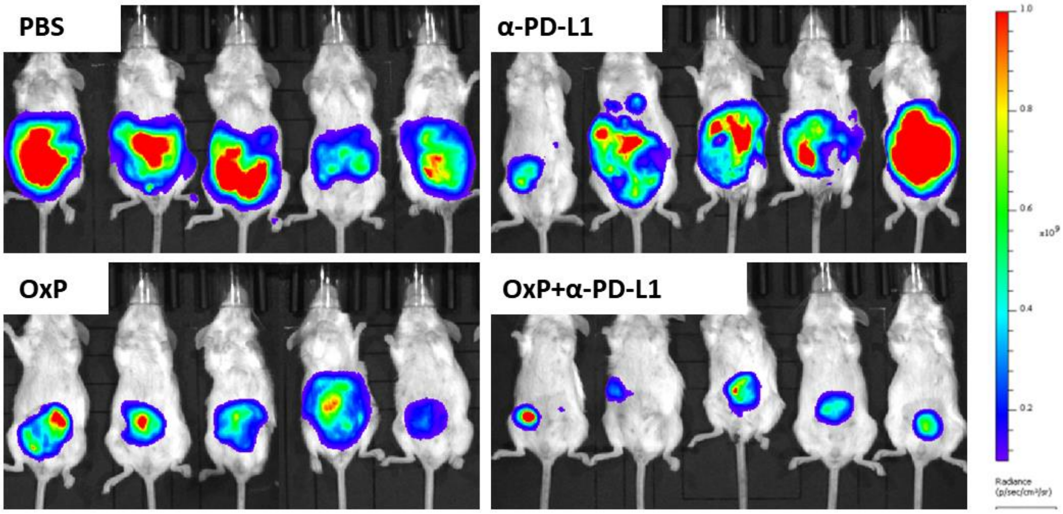

Supplementary Figure 4. IVIS images of the orthotopic CT26-FL3 tumor-bearing mice after various treatments on day 33.

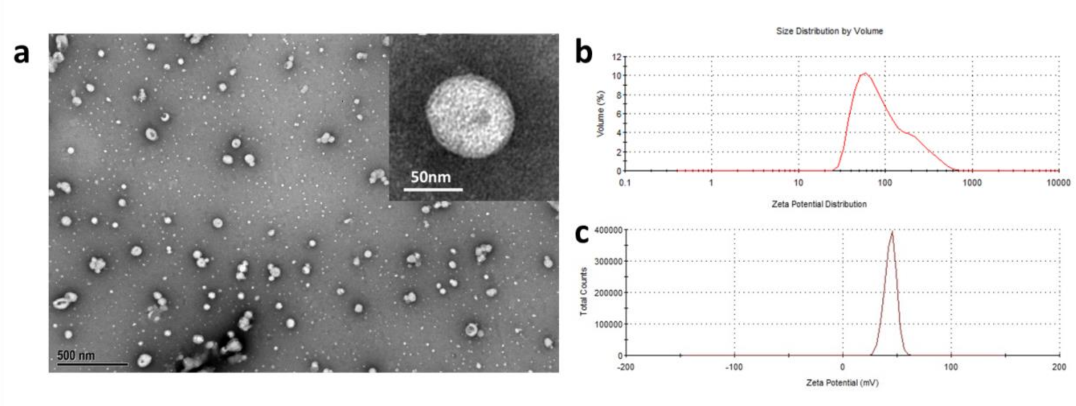

Supplementary Figure 5. Characterization of the prepared LPD-PD-L1 trap plasmid nanoparticles. **a** TEM images. **b** Volume average size detected by DLS. **c** Zeta potential.

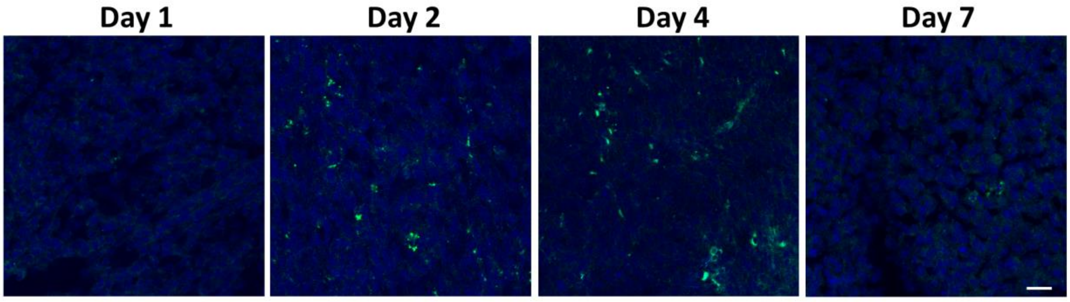

Supplementary Figure 6. GFP expression in tumor tissues on day 1, 2, 4 and 7. GFP plasmid loaded LPD nanoparticles were given on day 0 at a dose of 50 μg plasmid per mouse. Scale bar represents 20 μm.

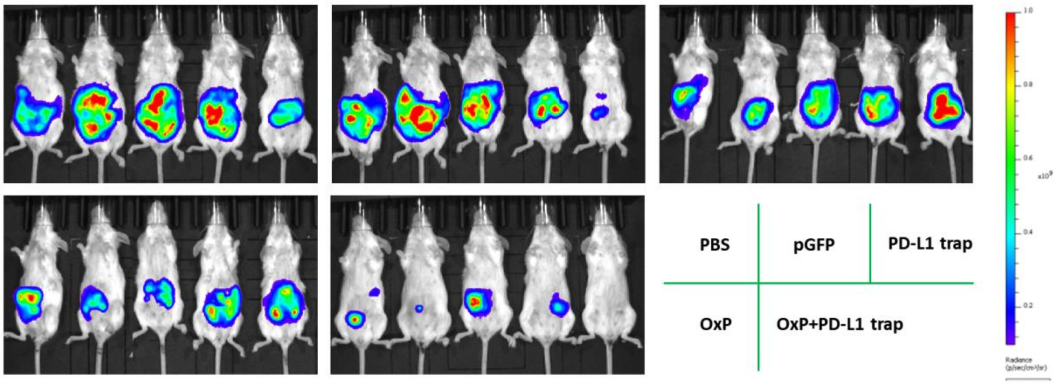

Supplementary Figure 7. IVIS images of the orthotopic CT26-FL3 tumor-bearing mice after various treatments on day 33.

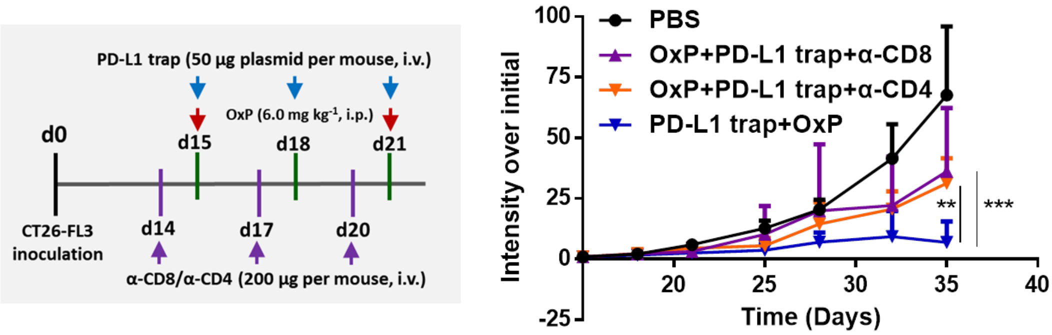

Supplementary Figure 8. Orthotopic CT26-FL3 tumor growth curves of the PBS, OxP+PD-L1 or combined with α-CD8/α-CD4 groups (n = 5 mice per group). Significant differences were assessed using two-way ANOVA. Results are presented as mean (SD). ** $P < 0.01$, *** $P < 0.001$.

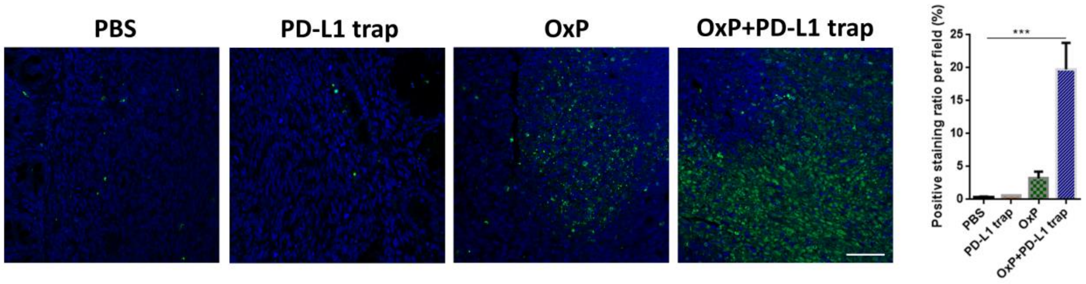

Supplementary Figure 9. TUNEL staining of the PBS, PD-L1 trap, OxP and OxP+PD-L1 trap treated CT26-FL3 tumor tissues on day 28. Positive ratios were quantified in 3 randomly selected fields per mouse (n = 4 mice per group). Scale bar represents 50 μm. Significant differences were assessed using t test. Results are presented as mean (SD). *** P < 0.001.

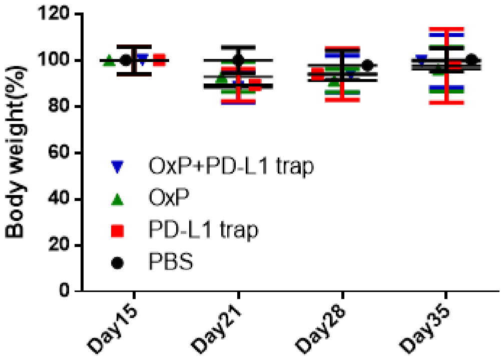

Supplementary Figure 10. Body weight over initial of the orthotopic CT26-FL3 tumor-bearing mice in PBS, PD-L1 trap, OxP and OxP+PD-L1 trap treated groups (n = 5).

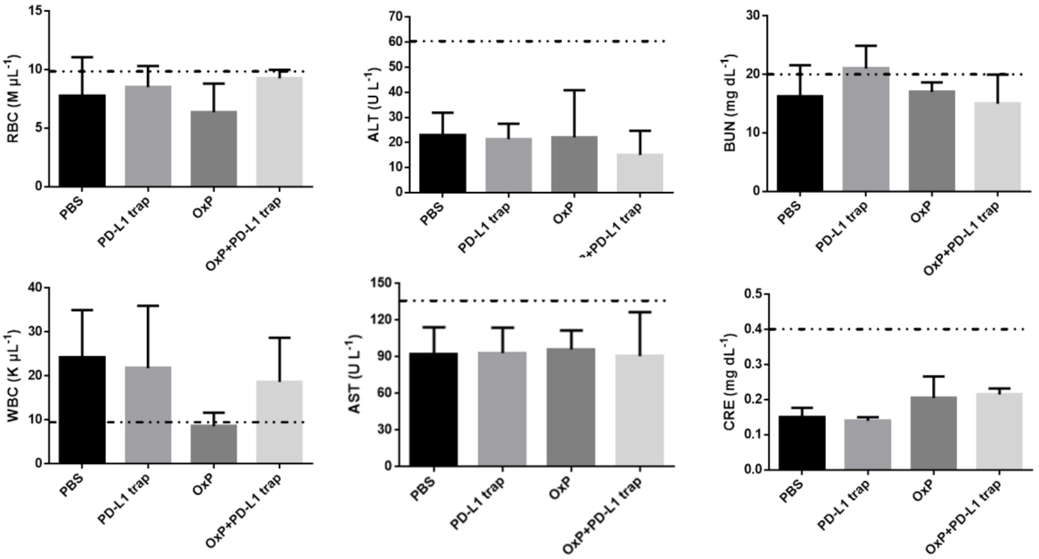

Supplementary Figure 11. Complete blood count and blood chemistry analysis of the orthotopic CT26-FL3 tumor bearing mice after various treatments. Measurements were carried out on day 28 (n = 3).

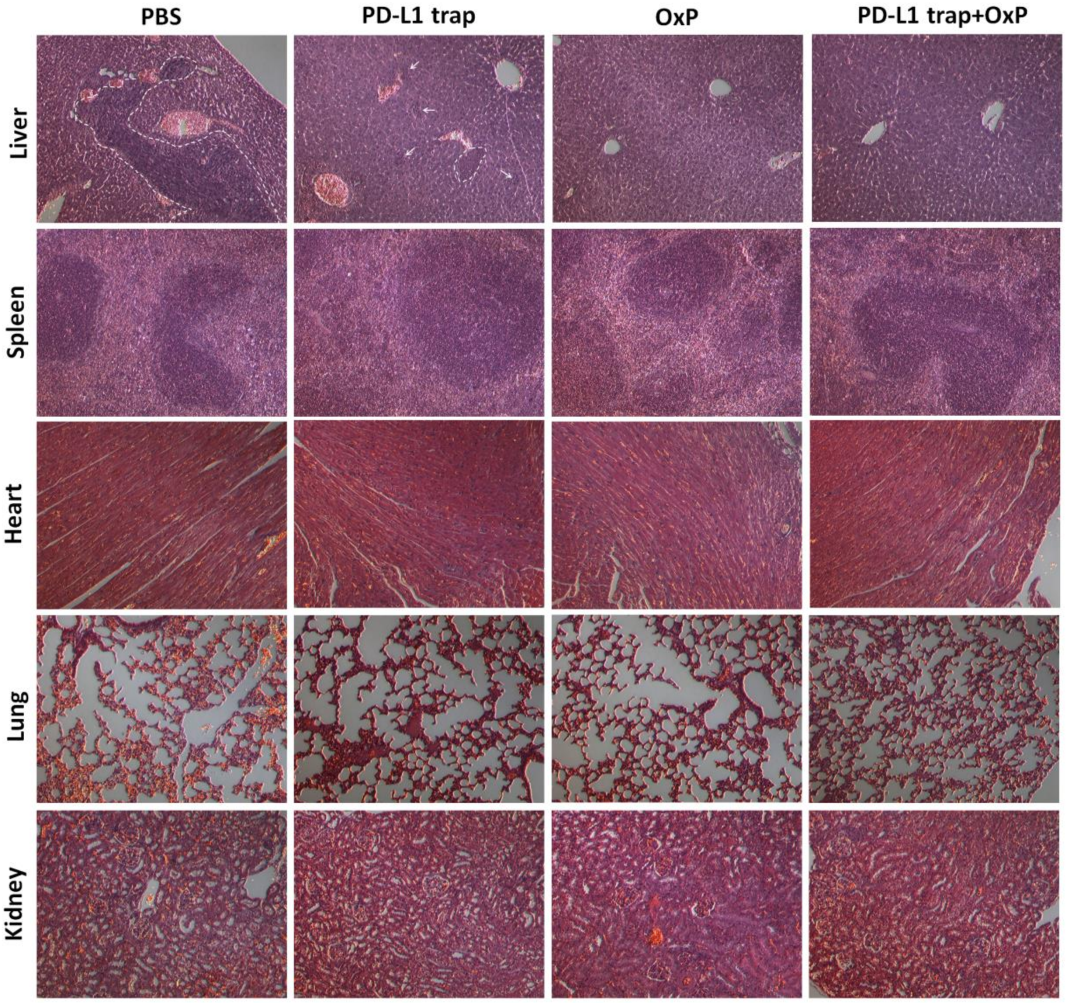

Supplementary Figure 12. Pathological analyses of liver, spleen, heart, lung and kidney of the CT26-FL3 tumor-bearing mice in the PBS, PD-L1 trap, OxP and OxP+PD-L1 trap treated groups on day 28. The regions in the white lines or sites pointed by white arrows in liver are metastasis sites. The photos were taken at 20 × magnification.

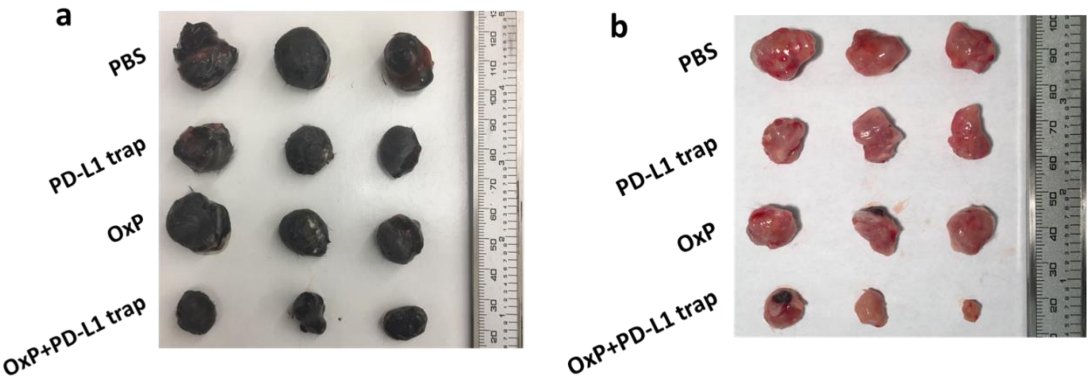

Supplementary Figure 13. Representative images of the B16F10 (**a**) and 4T1 (**b**) tumors at the end of observation.

Supplementary Tables

Supplementary Table 1. Antibodies used in the study.

Antibodies	Company	Catalog No.	Application	Dilution fold
Alexa Fluor® 488 Anti-CD45	BioLegend	103122	IF	200
Alexa Fluor® 647 Anti-CD3	BioLegend	100209	IF	200
Alexa Fluor® 594 Anti-CD11c	BioLegend	117346	IF	200
Alexa Fluor® 594 Anti-CD4	BioLegend	100446	IF	200
Alexa Fluor® 488 Anti-FoxP3	BioLegend	126406	IF	200
Alexa Fluor® 594 Anti-F4/80	BioLegend	123140	IF	200
FITC Anti-NK1.1	BioLegend	108706	IF	200
Alexa Fluor® 594 Anti-Gr1	BioLegend	108448	IF	200
Alexa Fluor® 488 Anti-CD11b	BioLegend	101217	IF	200
APC Anti-IL-10	BioLegend	505010	IF	200
Anti-HMGB1	Abcam	ab18256	IF	500
Anti-CRT	Abcam	ab2907	IF	500
Goat Anti-Rabbit IgG, Alexa Fluor® 594	Abcam	Ab150080	IF	500
Alexa Fluor® 594 Anti-CD8a	BioLegend	100758	Flow	500
PE Anti-CD206	BioLegend	141706	Flow	500
APC Anti-DC Marker	BioLegend	124913	Flow	500
APC-Cy7 Anti-CD3e	BioLegend	557596	Flow	500
BV605 Anti-CD274	BioLegend	329724	IF, Flow	200, 500
APC Anti-mouse IL-17A	BioLegend	506916	Flow	500
PerCP Anti-CD45	BD Pharmingen	550994	Flow	500
FITC Anti-CD4	Santa Cruz	D3010	Flow	500
Purified anti-human CD3	Biolegend	300301	IF, IHC	200
Donkey Anti-mouse IgG, Alexa Fluor® 594	Abcam	Ab150108	IF	200

IF: immunofluorescence. IHC: immunohistochemistry. Flow: Flow cytometry.

Supplementary Table 2. Primers used in this study

**Primer**	**Applied Biosystems**
Mouse GAPDH	Mm99999915_g1
Mouse IFN-γ	Mm01168134_m1
Mouse TNF-α	Mm00443260_g1
Mouse IL4	Mm00445259_m1
Mouse IL10	Mm01288386_m1
Mouse CXCL10	Mm00445235_m1
Mouse CXCL9	Mm00434946_m1
Mouse CCL2	Mm00441242_m1
Mouse CXCL12	Mm00445553_m1
Mouse CXCL13	Mm04214185_s1

Supplementary Note 1. Nucleic acid sequences coding the open reading frame of the mouse PD-L1 trap (*).

ATGAAATGGGTCACCTTTATCAGCCTGCTGTTCCTGTTCAGCAGCGCCTACTCTGGATC
CTGGCTGCTGGAAGTGCCTAACGGCCCTTGGAGAAGCCTGACATTCTACCCTGCCTGG
CTGACCGTGTCTGAGGGCGCTAACGCCACCTTCACATGCAGCCTGAGCAATTGGAGCG
AGGACCTGATGCTGAACTGGAATAGACTGAGCCCCAGCAACCAGACCGAGAAGCAGG
CCGCTTTCTGCAACGGACTGTCTCAGCCTGTGCAGGACGCCAGATTCCAGATCATCCA
GCTGCCTAACAGACACGACTTCCACATGAACATCCTGGACACCAGAAGAAACGACAG
CGGCATCTACCTGTGCGGCGCCATCTCTCTGCACCCCAAGGCTAAGATCGAGGAATCTC
CTGGCGCTGAGCTGGTGGTCACCGAGAGAATTCTTGAAGGCCCGCAACCGCAACCGA
AACCGCAGCCGAAACCGGAACCGGAACCGCAACCGCAAGGCGGTTCTGAGGAAGAC
CCCTGTGCCTGTGAGTCCATACTGAAATTTGAGGCCAAGGTGGAGGGTCTGCTGCAGG
CCCTGACCAGGAAGCTGGAAGCTGTGAGCGGGCGGCTGGCTGTCCTGGAGAACAGAA
TCATCGCGGCCGCTGGCGCCCCTGTGCCTTATCCTGATCCCCTGGAACCTAGAGGCGGC
AGCCACCACCACCATCACCACTGA

*: From PCT/US2016/051966
